# Use data augmentation for a deep learning classification model with chest X-ray clinical imaging featuring coal workers' pneumoconiosis

**DOI:** 10.1186/s12890-022-02068-x

**Published:** 2022-07-15

**Authors:** Hantian Dong, Biaokai Zhu, Xinri Zhang, Xiaomei Kong

**Affiliations:** 1 The First College for Clinical Medicine, Shanxi Medical University, No. 56 Xinjian South Road, Taiyuan, 030001 Shanxi People’s Republic of China; 2 Network Security Department, Shanxi Police College, No. 799 Qingdong Road, Qingxu Country, Taiyuan, 030021 Shanxi People’s Republic of China; 3 National Health Commission Key Laboratory of Pneumoconiosis, Shanxi Key Laboratory of Respiratory Diseases, Department of Respiratory and Critical Care Medicine, First Hospital of Shanxi Medical University, No. 85 Jiefang South Road, Taiyuan, 030001 Shanxi People’s Republic of China

**Keywords:** Coal workers' pneumoconiosis classification, Chest X-ray, Deep learning, ShuffleNet, ECA-Net, Data augmentation

## Abstract

**Purpose:**

This paper aims to develop a successful deep learning model with data augmentation technique to discover the clinical uniqueness of chest X-ray imaging features of coal workers' pneumoconiosis (CWP).

**Patients and methods:**

We enrolled 149 CWP patients and 68 dust-exposure workers for a prospective cohort observational study between August 2021 and December 2021 at First Hospital of Shanxi Medical University. Two hundred seventeen chest X-ray images were collected for this study, obtaining reliable diagnostic results through the radiologists' team, and confirming clinical imaging features. We segmented regions of interest with diagnosis reports, then classified them into three categories. To identify these clinical features, we developed a deep learning model (ShuffleNet V2-ECA Net) with data augmentation through performances of different deep learning models by assessment with Receiver Operation Characteristics (ROC) curve and area under the curve (AUC), accuracy (ACC), and Loss curves.

**Results:**

We selected the ShuffleNet V2-ECA Net as the optimal model. The average AUC of this model was 0.98, and all classifications of clinical imaging features had an AUC above 0.95.

**Conclusion:**

We performed a study on a small dataset to classify the chest X-ray clinical imaging features of pneumoconiosis using a deep learning technique. A deep learning model of ShuffleNet V2 and ECA-Net was successfully constructed using data augmentation, which achieved an average accuracy of 98%. This method uncovered the uniqueness of the chest X-ray imaging features of CWP, thus supplying additional reference material for clinical application.

**Supplementary Information:**

The online version contains supplementary material available at 10.1186/s12890-022-02068-x.

## Introduction

Coal workers' pneumoconiosis (CWP) is an occupational lung disease whose specific pathological changes include diffuse interstitial lung fibrosis [1] due to prolonged exposure to excessive quantities of respiratory coal dust. This condition can lead to irreversible and potentially fatal lung diseases, including chronic obstructive pulmonary disease, tuberculosis, chronic bronchitis, emphysema, and other lung diseases [2]. Although the prevalence of CWP has substantially decreased over the past few decades, according to recent research [2–4], the incidence in the eastern region of the United States and the state of Queensland in Australia has shown a worryingly increasing trend. In China, the prevalence of CWP appears high, with one report indicating that it constituted over 6% of all Chinese occupational diseases in the 2010s; specifically, over 13,000 miners were diagnosed with CWP in 2013 and 2014 [5]. Moreover, according to the China National Report of occupational diseases, CWP was one of the most prevalent types of pneumoconiosis among all age groups, and most pneumoconiosis cases were diagnosed among males [6].

Although pneumoconiosis is prevalent and there are no effective therapies for the treatment of CWP, an early diagnosis can broadly prevent the development of complications and aid in the development of early treatments for any that do arise. Currently, X-ray is the essential tool for suggesting whether there are any suspicious findings of pneumoconiosis via the identification of subtle graphic patterns and features described in the International Labour Organisation (ILO) guidelines. The typical radiological features of CWP include small nodular interstitial opacities in the upper zones; however, not all these features are exclusive to CWP [7]. A long-term evaluation of CWP in America found that almost 40% of coal miners with radiographical interstitial changes had predominantly irregular opacities [8]; on X-ray images, these irregular opacities may also be features of mixed-dust pneumoconiosis (MDP), which, pathologically, manifests as a pneumoconiosis showing dust macules or mixed-dust fibrotic nodules, with or without silicotic nodules, in an individual with a history of exposure to mixed dust [9]. The radiological findings of CWP with secondary lung disease might be similar to those seen in idiopathic pulmonary fibrosis [7, 10]. In addition, the respiratory symptoms of CWP are nonspecific and mostly overlap with other coal dust-related conditions, such as chronic bronchitis, chronic obstructive pulmonary disease (COPD), and emphysema [11]. Many CWP and dust-exposed workers often choose general hospitals for treatments when they begin coughing and presenting with dyspnoea. Generally, the imaging workup for chest diseases starts with a chest X-ray (CXR), but it has a limited role in diagnosing pulmonary complications of pneumoconiosis because of overlapping pneumoconiotic infiltration [12]. Patients with CWP experience many complications, such as chronic interstitial pneumonitis and pneumothorax; hence, knowledge of the CWP imaging features on chest radiographs are important for improving the rates of early diagnosis and cure, especially considering the difficulties in recognizing and treating the disease.

Prior studies have reported encouraging results in medical image analysis with artificial intelligence (AI) [13–18]. Although they are unlikely to replace radiologists for the foreseeable future, AI algorithms have achieved performance comparable to that of radiology experts in interpreting CXRs [19]. Deep learning (DL), a subdiscipline of AI, has emerged as a new solution for many medical image analysis problems, with remarkable success in classifying pneumoconiosis grade and exploring the application of AI in detecting pneumoconiosis [20, 21]. The merits of DL lie in its ability to learn complex imaging features or patterns inconspicuously without purposefully identifying and extracting them, as tens of millions of features may be involved and analysed to obtain high-level features [18].

Many kinds of deep learning models, also known as deep convolutional structures, such as EfficentNet [22], VGGNet [23], ResNet [24], ZDNet [25], DenseNet [26], and GoogleNet [27], have been proposed that can provide peer-to-peer solutions for image feature extraction and are superior to traditional criteria in almost all image recognition tasks [13]. In the field of pneumoconiosis screening and staging, the core design of these experiments mainly depended on massive datasets. Challenged by unadequate analysis of limited data, to address this problem, it is necessary to apply data augmentation to increase the additional data in our deep learning model, which could help DL model more generalisable and ultimately improve performance on the model training.

In this paper, we proposed a deep learning classification model based on the data augmentation technique that could steadily improve the ability of radiologists to interpret CWP chest radiographs by screening and discovering the uniqueness of imaging features of CWP, thus potentially improving the comprehension of the clinical radiologic manifestations of pneumoconiosis.

## Materials and methods

### Patients and data collection

Patients with CWP or who were exposed to dust who voluntarily participated in the Coal Mining Workers' Pneumoconiosis and dust exposure Cohort Study (CONDUCT) between 28 August 2021 and 12 December 2021 were enrolled. CONDUCT was a CWP-based prospective cohort study conducted to understand the epidemiological characteristics of CWP, to explore the related risk factors for pneumoconiosis in addition to dust and to encourage research on CWP in terms of early diagnosis, novel mechanisms and drug discovery.

In the study reported here, we collected workers with confirmed CWP and dust-exposed workers from coal mines around Taiyuan City complicated with cough, dyspnoea, or other symptoms. Most of the patients were initially treated at a local tertiary-A integrated hospital for CXR assessment. Among the coal mining workers with the chronic respiratory symptoms listed above, we identified those who participated in CONDUCT after obtaining permission from the Taiyuan City Center for Disease Control and Prevention and provided them with yearly clinical systematic health checks. We classified the imaging features listed in the corresponding examination reports and extracted features from the target regions of the pneumoconiosis CXR images using the enhanced ShuffleNet V2 combined with ECA-Net based on deep learning. This model was used for target region classification training to perform clinical imaging feature classification for CWP/dust-exposed workers and to explore the application of deep learning in imaging feature detection for CWP.

All CXRs analysed in our study were collected from the first of three yearly follow-up surveys from CONDUCT in 2021. All patients included in this study signed informed consent forms, and the Ethics Committee of the First Hospital of Shanxi Medical University approved the study (2020 K-K104). The database established by the cohort study was registered in the Chinese Clinical Trial Registry (27/08/2021, ChiCTR2100050379). It included 217 anonymized CXRs, from male patients aged 35–80 years with cough, dyspnoea, or other symptoms; 149 patients were placed in the CWP group (whose stages are defined in Table 1), and the remaining 68 patients were placed in the standard group (dust-exposed workers). For patients with multiple previous hospitalizations or visits, the data during this study were adopted. The duration of exposure to dust (for patients with a clear dust-exposure history) was more than 15 years, with an average of 28.95 ± 14 years. We present the patient baseline characteristics of the different groups in Table 2.

**Table 1:** Definition of CWP stages (according to GBZ70-2015)

	Summary of pneumoconiosis standard (CXR)
Dust-exposure workers (Coal Mining Workers with clear dust-exposure history)	No opacities discovered, or small opacities (Level 1 profusion) discovered in one subregion
CWP Stage I	Small opacities (Level 1 profusion) discovered in two subregions at least, or small opacities (Level 2 profusion)discovered in four subregions at most
CWP Stage II	Small opacities (Level 2 profusion) discovered in four subregions at least, or small opacitie (Level 3 profusion) discovered
CWP Stage III	Small opacities (Level 3 profusion) discovered in four subregions at least, or large opacities discovered

**Table 2:** Baseline characteristics

	All subjects	Dust-exposed workers	CWP Stage I	CWP Stage II	CWP Stage III
			M (SD)		
Age (yr), mean (SD)	55 (13)	48.7 (6.0)	60 (11)	57.9 (7.9)	56.3 (7.6)
Exposure duration (yr), mean (SD)	29.0 (14)	21.6 (7.5)	32.0 (10)	27.0 (14)	16.4 (1.5)
Dyspnoea (SD)	3 (9)	0 (3)	6 (9)	4 (11)	3.7 (2.1)
Cough (SD)	0.3 (6)	0 (3)	2 (9)	0 (7)	8.3 (4.6)
mMRC score (SD)	2 (2)	0 (2)	2 (2)	2 (0)	1.8 (0.8)
*Industry type*					
Total	217	63	130	22	2
Mining (n)	63	32	26	5	0
Tunnelling (n)	105	29	60	14	2
Comprehensive digging (n)	17	0	16	1	0
Mixing (n)	19	2	15	2	0
Other (n)	13	0	13	0	0

M: mean, SD: standard deviation, mMRC: modified Medical Research Council

### Chest X-ray acquisition

We obtained chest radiographs using a mobile X-ray system (Carestream, DRX-Revolution VX3733-SYS, America), with a tube voltage of 120–150 kVp, exposure time of 100 ms, and optimum source-to-image distance (SID) of 180 cm. After calibration, the industry standard regulated by Digital Imaging and Communications in Medicine (DICOM) was required for the CXR images.

### Classification and segmentation

To build the CWP CXR imaging feature classification system, we invited three radiologists with 5–10 years of experience in interpreting CXR to create imaging diagnosis reports for all CXRs, which were subsequently independently analysed. Then, two senior radiologists with over 10 years of experience reviewed these diagnostic results and made the final diagnostic decisions. During this period, the senior radiologists were not informed of any details of the CWP study.

**Fig. 1:**
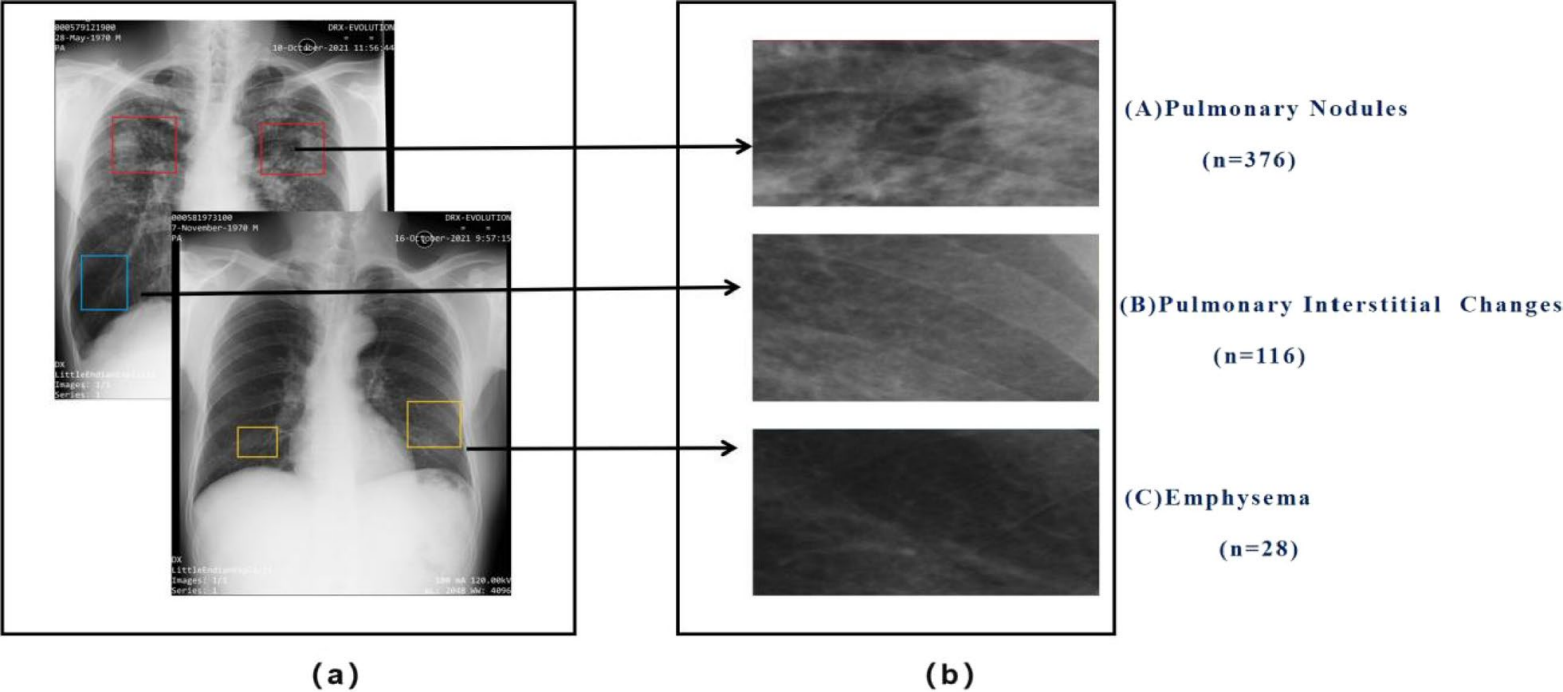
Original CXR **a** with identifying target lung region. We segmented **b** regions of interest (ROIs) classified into three types

The clinical CXR imaging features were confirmed based on the diagnostic reports by our experienced radiology team and included the following: (A) pulmonary nodules; (B) pulmonary interstitial changes; (C) emphysema; (D) pleural plaques; and (E) mediastinal masses. Previous research on pneumoconiosis classification proposed handcrafted feature extraction from each region of interest (ROI) [28, 29]. To identify the target lung region in each CXR, we segmented the ROIs (shown in Fig. 1) from the lung field via screenshot while referencing the diagnosis reports. Moreover, these images were saved as JPEG images of appropriate size (at least 30 × 30 pixels). Of the 217 patients recruited in the study, one ROI was segmented from 171 patients each (171 ROIs) two ROIs were segmented from 22 patients each (44 ROIs), and three ROIs were segmented from the remaining 24 patients each (72 ROIs). The ROIs were then assigned the following data labels: pulmonary nodules (n = 376), pulmonary interstitial changes (n = 116), and emphysema (n = 28); pleural plaques and mediastinal masses were excluded, as they were severely underrepresented on the CXRs (n < 10). Finally, all imaging features were classified into three categories: A (pulmonary nodules), B (pulmonary interstitial changes), and C (emphysema) (shown in Fig. 1). Table 3 shows the detailed distribution of the clinical CXR imaging features.

**Table 3:** Distribution of clinical CXR imaging features

CXR Imaging feature	Lung zone	Number of ROIs
Pulmonary nodules	Top-right	83
	Middle-right	77
	Bottom-right	26
	Top-left	112
	Middle-left	55
	Bottom-left	23
	Total	376
Pulmonary interstitial changes	Top-right	2
	Middle-right	21
	Bottom-right	24
	Top-left	1
	Middle-left	33
	Bottom-left	35
	Total	116
Emphysema	Top-right	8
	Middle-right	3
	Bottom-right	4
	Top-left	6
	Middle-left	4
	Bottom-left	3
	Total	28

ROI: Region of interest

### Deep learning CWP image data augmentation model

Due to the limitations of small datasets, Devnath et al. [30] first proposed transfer learning with a convolutional neural network (CNN) for the detection of pneumoconiosis disease on CXR. Subsequently, many studies [31, 32] confirmed CWP detection on CXR using data augmentation, which improved the quality of the deep CNN, increased the amount of training data, and outperformed other statistical and traditional machine learning approaches as well as radiologists. Considering the particular characteristics of medical images, we performed some additional information or data transformation on the original images to selectively highlight specific regions or suppress unimportant regions, thus enhancing the image data, making them more conducive to model training and avoiding overfitting the results of the model.

**Fig. 2:**
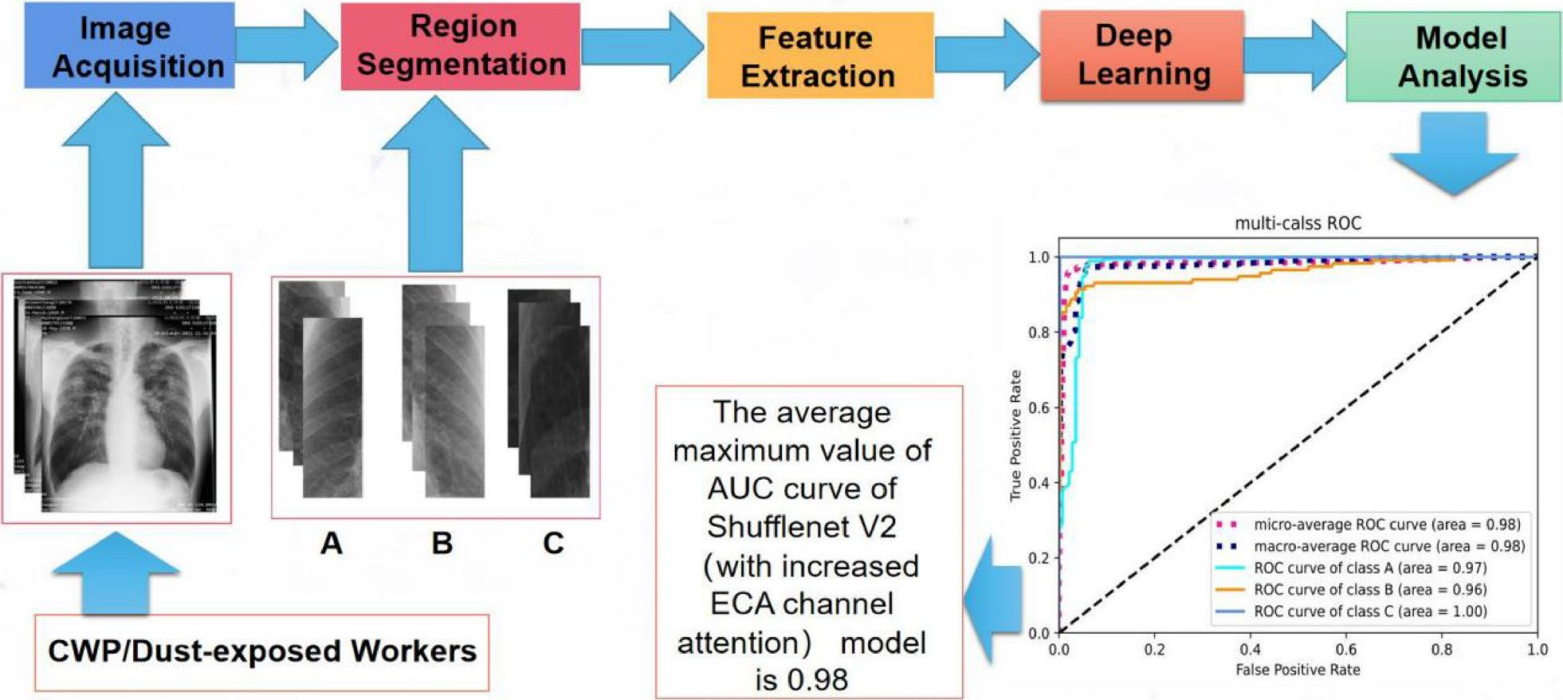
Flowsheet clarifying the procedure of classifying CXR clinical features among CWP

**Fig. 3:**
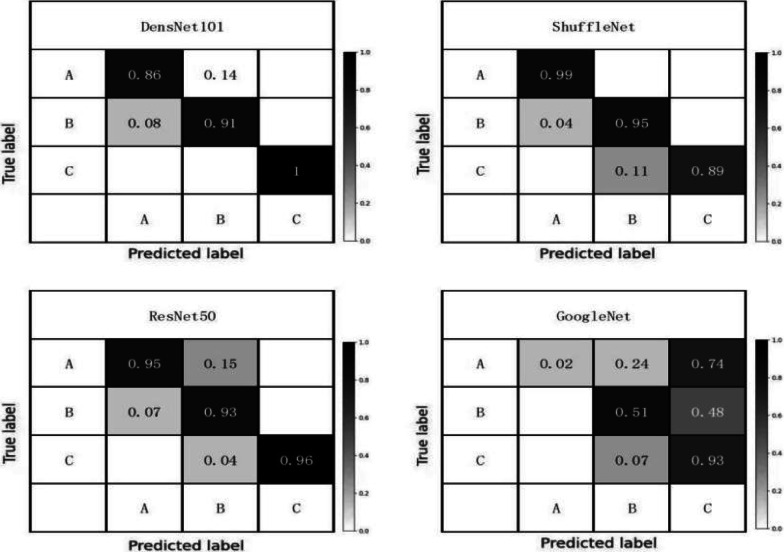
Comparison of accuracy in CWP classification with different algorithms

The flow chart of the method used in this study is shown in Fig. 2, and the confusion matrix of the accuracy of multiple models in Fig. 3. In this study, through comprehensive comparisons, the original ShuffleNet model was shown to possess higher classification accuracy and recall than the other models; thus, we chose it for modification to further improve its performance.

### ShuffleNet

Practical integration of data augmentation is an important discussion point regarding the development of future deep learning workflows [33, 34]. As our dataset contained 520 ROIs from a total of 217 images, it was essential to provide an adequate analysis of the limited data. ShuffleNet is an exceedingly computationally efficient CNN model proposed by Zhang X in 2018, who designed this new model to perform two new operations, pointwise group convolution and channel shuffling, to significantly reduce the computational cost while maintaining accuracy [35]. Compared with ImageNet classification and MSCOCO object detection, their study demonstrated the superior performance of ShuffleNet over other structures and highlighted the advantages of packet convolution in operational efficiency, especially for small datasets.

### Efficient channel attention (ECA)-Net

Some researchers have claimed that a multiscale augmentation strategy is crucial for data expansion and thus increasing the accuracy of classification modelss [20]. The channel attention mechanism has been proven to improve the accuracy of CNN models; however, the performance improvement achieved by incorporating such sophisticated attention methods unavoidably increases the model complexity. In 2020, Wang et al. [36] proposed an efficient channel attention (ECA) module that only requires a handful of parameters while providing apparent performance improvements. The ECA-Net mechanism is a “plug-and-play”, lightweight attention module that generates channel attention by using fast 1D convolution to ensure model efficiency and accuracy. Considering the superiorities of ECA-Attention, it can lead to significant model accuracy improvements despite the use of finite sample data, thus increasing the ability to classify imaging features.

### Image data augmentation

Current studies [37, 38] suggest that image data augmentation could be divided into two major categories: basic image manipulations and augmentation methods based on deformable techniques and DL-based approaches.This study applied augmentation technique based on basic image manipulation. To expand the ROI dataset and increase the robustness of the model, all ROIs were segmented manually, despite the potential lack of objectivity; it was necessary to perform image data augmentation in case of overfitting during the training process, which we summarize as follows. First, we performed k-fold cross-validation to split the original dataset into three groups: the training group accounted for 70% of the data, the validation group accounted for 20%, and the test group accounted for 10%. Second, ROIs were randomly selected from different groups in the original dataset and processed with all kinds of image dataset augmentation techniques (shown in Table 4). These operations yielded a total of over 12,000 training images were acquired.

**Table 4:**
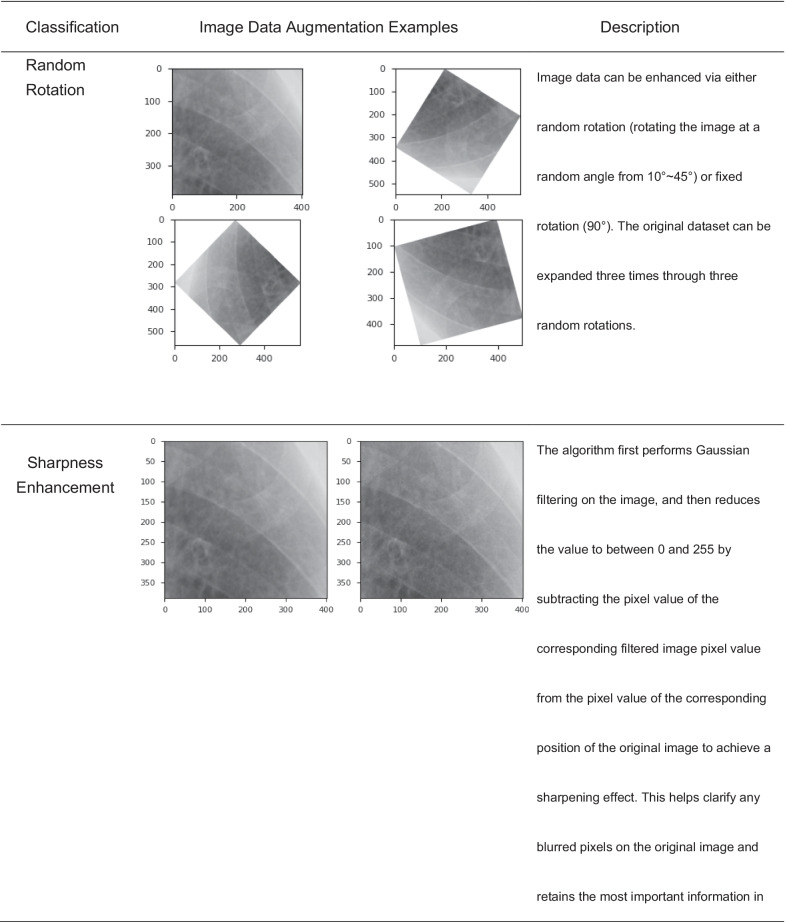

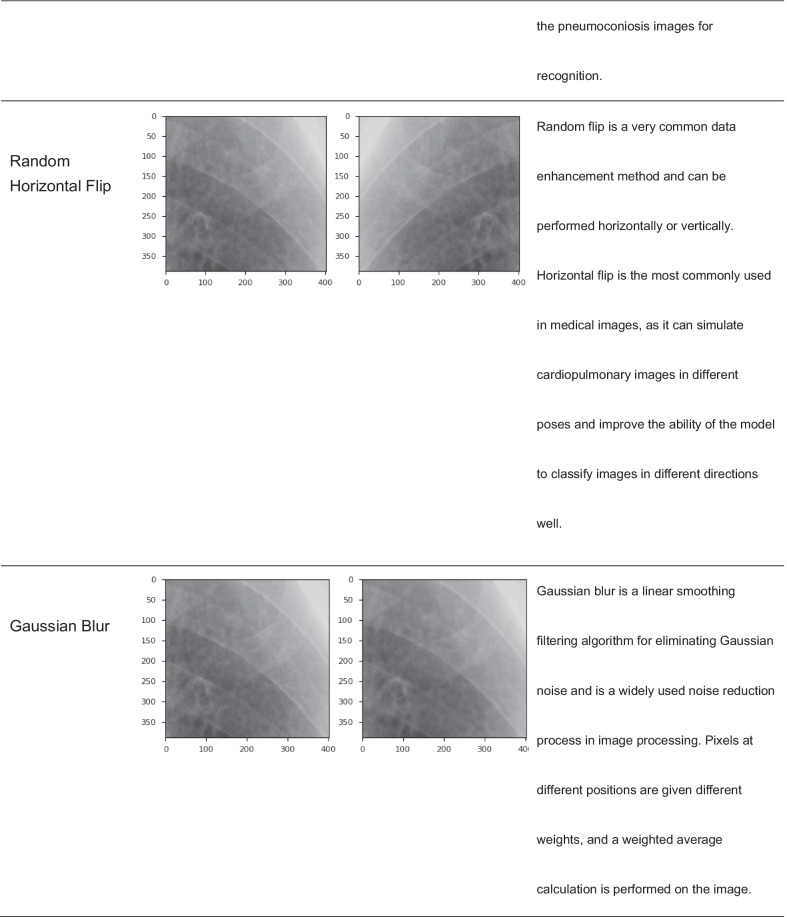

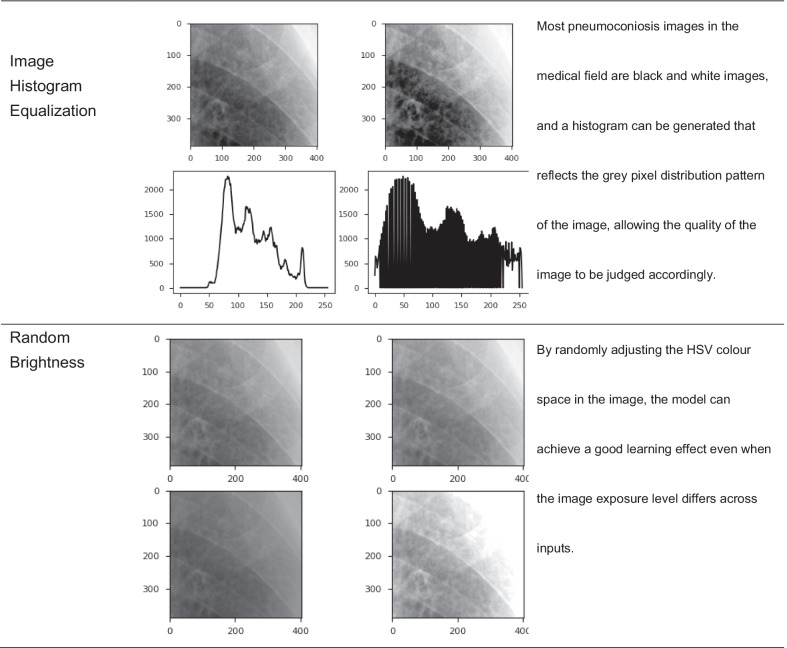
Detailed classification of image dataset augmentation

### Implementation details

All ROIs were divided into ten groups (ten-fold cross-validation); the individual groups were separately adopted as test data, and the remaining nine groups were used for training in turn; thus, each round trained 16 images. We set the initial learning rate to 0.0001, the number of training epochs to 30, and the classification output to three types. Through the PyTorch tool, the above steps could be performed adequately with one RTX 2080Ti GPU.

### ShuffleNet V2 combined with ECA-Net (ShuffleNet-Attention)

The ShuffleNet-v2 network is an optimized version the ShuffleNet structure based on criteria such as optimal MAC, reduced network fragmentation, and reduced elementwise operations [39]. Several preliminary experimental results showed that using the original ShuffleNet for classification training always confused pulmonary interstitial changes with emphysema. To avoid causing feature misidentification, we chose ShuffleNet V2 as the model backbone and combined it with the ECA-Net mechanism after each convolution layer, creating ShuffleNet-Attention (Additional fles 1, 2, 3, 4). Through this combination of two models, the convolution layers of each block can be simplified, while more semantic information is conveyed to the feature layers. This reduced the computational complexity by more than 40% with respected the original network, markedly increasing the classification accuracy (from 92 to 94%) more rapidly.

### Evaluation metrics

As performance metrics for the model, accuracy, loss function, precision, recall, F1-score, the receiver operating characteristic (ROC) curve and the area under the ROC curve (AUC) were evaluated. The evaluation metrics involved in this study are calculated as follows:

1 $${\text{Accuracy}} = \frac{{{\text{Total}}\,{\text{of}}\,{\text{all}}\,{\text{true}}\,{\text{classsification}}}}{{{\text{Total}}\,{\text{of}}\,{\text{all}}\,{\text{images}}\,{\text{classsification}}}}$$

Accuracy is defined here as the number of correctly classification of CWP clinical features divided by the total number of classification of CWP clinical features evaluated. In deep learning classifcation tasks, the loss function is commonly applied to optimize the parameters of the model, which can improve the classifcation accuracy and promote the weights of categories with smaller samples effectively.

2 $${\text{Recall}} = \frac{{{\text{Total}}\,{\text{of}}\,{\text{all}}\,{\text{false}}\,{\text{positive}}\,{\text{classsifications}}\,{\text{(FP)}}}}{{{\text{Total}}\,{\text{of}}\,{\text{all}}\,{\text{false}}\,{\text{positive}}\,{\text{classsifications}}\,{\text{(FP)}} + {\text{Total}}\,{\text{of}}\,{\text{all}}\,{\text{true}}\,{\text{negative}}\,{\text{classsifications}}\,{\text{(TN)}}}}$$

Recall is defined here as the proportion of correctly classification of CWP clinical features.

3 $${\text{Precision}} = \frac{{{\text{Total}}\,{\text{of}}\,{\text{all}}\,{\text{true}}\,{\text{positive}}\,{\text{classsifications}}\,{\text{(TP)}}}}{{{\text{Total}}\,{\text{of}}\,{\text{all}}\,{\text{true}}\,{\text{positive}}\,{\text{classsifications}}\,{\text{(TP)}} + {\text{Total}}\,{\text{of}}\,{\text{all}}\,{\text{false}}\,{\text{positive}}\,{\text{classsifications}}\,{\text{(FP)}}}}$$

The precision measures the probability of making correct positive classification of CWP clinical features.

4 $${\text{F1 - score}} = \frac{{{2} \times {\text{Precision}} \times {\text{Recall}}}}{{{\text{Precision}} + {\text{Recall}}}}$$

A high F1-score reflects good classification performance, as it is the harmonic mean of precision and recall.The F1 score reaches its highest value at 1 and his lowest value at 0.

In order to help correctly evaluating the DL-model classifier integral performance, we evaluated the classified accuracy of CWP clinical features according to the receiver operating characteristic (ROC) curve and the area under the ROC curve (AUC).The ROC curve, which has been widely used in the field of medicine to evaluate the performance of deep learning diagnostic methods, is the plot of the true positive rate against the false negative rate at different threshold values, and the AUC is the area under the ROC curve and represents the quality of the DL model; a larger value indicates a better classification effect.

## Results

**Fig. 4:**
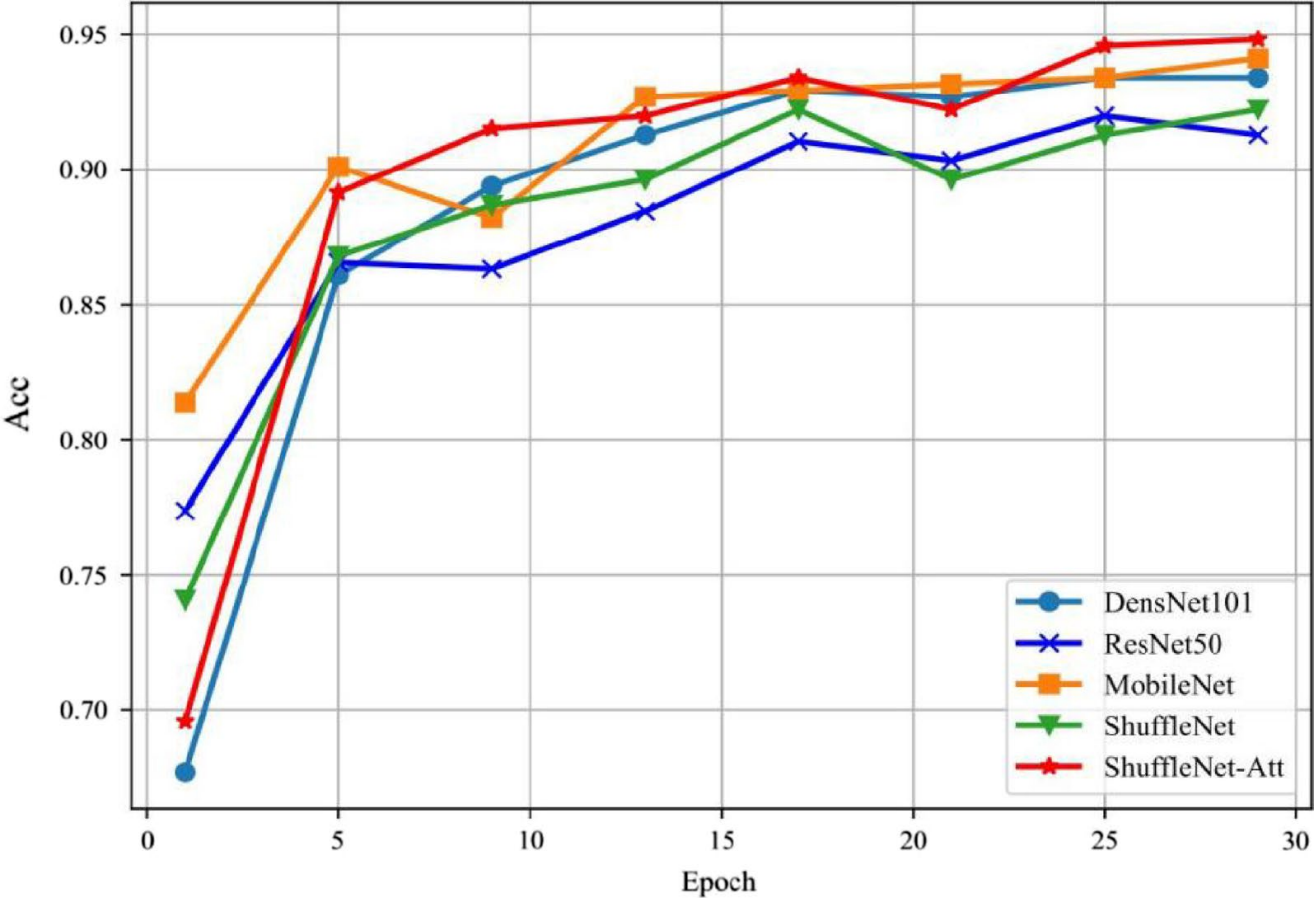
The accuracy in classification with different models with epochs

**Fig. 5:**
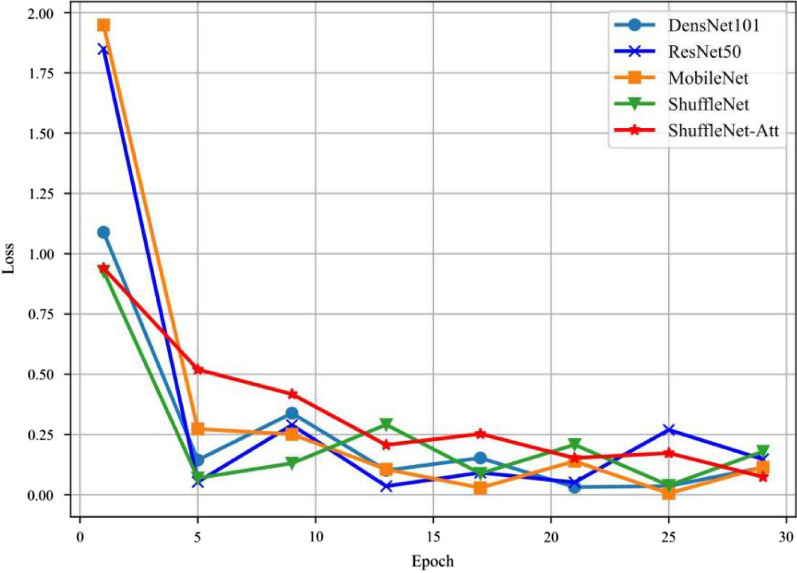
The losses in classification with different models with epochs

We summarized the model evaluation results using the test dataset (Table 5), the accuracy in CWP imaging feature classification with different models (Fig. 3), the accuracy versus the training epochs (Fig. 4), and the model loss versus the epochs (Fig. 5). ShuffleNet V2 combined with ECA-Attention (ShuffleNet-Att) reached the minimum value between epochs five and ten, slightly lower than the other models (Fig. 4). With sufficient training, the accuracy increased significantly after epoch 15, reaching more than 95% after epoch 25, while the loss curve (Fig. 5) reached its lowest point after epoch 25; thus, ShuffleNet-Attention ultimately achieved the best performance among these models.

**Table 5:** Performance of ShuffleNet v2, ResNet 50, GoogleNet, DenseNet 121 and ShuffleNet-Attention on the test set

	Accuracy	Recall	F1-score	Precision		
				Class A	Class B	Class C
Shufflenet-Attenion	0.96	0.97	0.96	0.96	0.92	0.93
Shufflenet v2	0.94	0.95	0.95	0.95	0.89	0.9
Resnet 50	0.9	0.94	0.93	0.93	0.85	0.84
Googlenet	0.91	0.94	0.92	0.92	0.91	0.82
Densnet 121	0.89	0.9	0.86	0.86	0.82	0.88

Class A: pulmonary nodules, Class B: pulmonary interstitial changes, Class C: emphysema

**Fig. 6:**
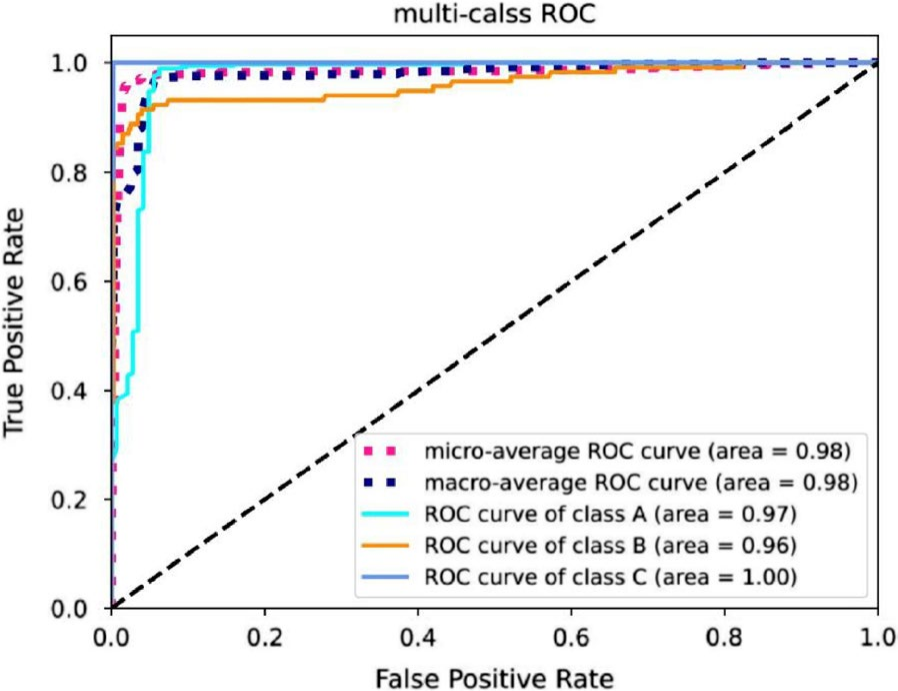
The ROC curve in classification with different models with epochs. Class A: pulmonary nodules, Class B: pulmonary interstitial changes, Class C: emphysema

Therefore, we selected ShuffleNet-Attention as the optimal model following this comparison and analysis. According to ROC curve analysis, the average AUC was 0.98; more specifically, the model achieved an AUC of 0.97 in classifying pulmonary nodules (Class A), 0.96 in classifying pulmonary interstitial changes (Class B), and 1.0 in classifying emphysema (Class C), as demonstrated in Fig. 6.

## Discussion

In the present study, we built a reliable model for classifying pneumoconiosis clinical imaging features by utilizing potential deep learning algorithms and well-annotated chest radiographs. To identify the clinical CXR imaging features of pneumoconiosis patients and dust-exposed workers more quickly and effectively, a computer-aided classification system was constructed by combining ShuffleNet V2 with ECA-Net.

Several previous studies have shown satisfactory performance for radiologists in using automatic DL-based models for pneumoconiosis screening and staging with CXR images. Among these studies [18, 21], the good performances were primarily attributed to the size of the datasets, which contained approximately 2000 chest radiographs from multiple centres or devices. When comparing the performance with evaluation metrics for different DL algorithms in interpreting pneumoconiosis, among a range of options available, the studies selected the best one to learn the image features to obtain an accurate classification of the pneumoconiosis grade. It is still necessary to obtain larger datasets whose lesion appearances of interest vary significantly for deep learning, implying that massive datasets play a vital role in these studies. In another study by Zhang et al.[20], a total of 405 DR images were analysed for screening and staging pneumoconiosis. They performed extensive image augmentation with a limited amount of training data, which vastly reduced the influence of sampling condition, image contrast, and lung size and improved the accuracy of the model. However, unlike the above studies, our study focused specifically CWP and aimed to analyse its imaging features from the perspective of secondary prevention to further improve the understanding of the disease in a clinical environment. In addition, CWP is the most common pneumoconiosis among dust-exposed workers; however, it is an entirely different disease from other pneumoconioses, such as silicosis. Even though their imaging features seem similar, there are considerable differences between CWP and other pneumoconiosis in terms of pathological characteristics, which makes specifically identifying CWP cases a challenge. We consider it essential to analyse and explore essential variables based on clinical knowledge of CWP.

This study focuses on classifying pneumoconiosis clinical imaging features for the following reasons: (1) Instead of screening and staging pneumoconiosis by only analysing lung regions on chest radiographs, it may be more necessary and useful to focus on analysing the characteristics of CWP with clinical eyes and classify pneumoconiosis clinical imaging features, as this may be more beneficial to the differential diagnosis of CWP and thus the early evaluation of the condition and the administration of early treatment. (2) Due to the complexity of the multiple classifications in pneumoconiosis and the particularity of the diagnosis process, workers exposed to coal minerals have an increased risk of CWP. The prediction models have high accuracy and were augmented with improvements in data extraction by deep learning techniques that include more clinically significant variables.

This study focuses on classifying pneumoconiosis clinical imaging features for the following reasons: (1) Instead of screening and staging pneumoconiosis by only analysing lung regions on chest radiographs, it may be more necessary and useful to focus on analysing the characteristics of CWP with clinical eyes and classify pneumoconiosis clinical imaging features, as this may be more beneficial to the differential diagnosis of CWP and thus the early evaluation of the condition and the administration of early treatment. (2) Due to the complexity of the multiple classifications in pneumoconiosis and the particularity of the diagnosis process, workers exposed to coal minerals have an increased risk of CWP. The prediction models have high accuracy and were augmented with improvements in data extraction by deep learning techniques that include more clinically significant variables.

Our study has several limitations. First, this sample size might be too small to obtained more detailed features for deep learning. We attempted to define the clinical difference between dust-exposed workers and patients with different CWP stages by classifying various imaging features. However, the sample size was 217 patients, which may have been too low. While this study had sufficient power to discern classifying outcomes correctly among these groups, more sample sizes would be needed to verify the accuracy of our study in the future. Second, the noninclusion of specific imaging features in the DL model, such as the position of pleural plaques and mediastinal masses, was not analysed, as the algorithm was not specifically trained for those features due to their low incidence in our dataset. A pooled analysis using the data from these features may be more beneficial in expanding the knowledge for different aspects of CWP clinically.Third, compared with the pulmonary nodules and pulmonary interstitial changes dataset, it is possible that the emphysema dataset was not sufficiently large to demonstrate significant feature differences, which might have contributed to the high accuracy for this classification. Shorten C et al. suggested that data augmentation cannot overcome all biases emerging in a small dataset; however, it can prevent overfitting by modifying limited datasets to better represent the characteristics of big data [33], which implies the need to obtain more imaging data for verification. After all, there are many limitations in identifying emphysema imaging features with chest radiographs. Computed tomography (CT) could provide more specific emphysema imaging features and should be analysed in future studies.

In addition, the clinical diagnosis of pneumoconiosis in China may be challenging. Hence, we believe that it remains crucial to better manage affected patients and to analyse valid clinical data. There may still be a lack of well-understood relationships between pneumoconiosis and imaging features on chest radiographs, and further research is needed.

## Conclusion

In this study, we classified CXR clinical imaging features of CWP using a deep learning technique on a small dataset, and a data augmentation model was successfully constructed by combining ShuffleNet V2 and ECA-Net. ShuffleNet-Attention demonstrated the best performance among the different models investigated and achieved an average accuracy of 98% for the imaging dataset. While this proposed method is incapable of screening and staging pneumoconiosis, the successful data augmentation model could assist radiologists in discovering the uniqueness of imaging features of CWP by using chest radiographs, thus supplying more references for clinical application.

Our study was part of a multicentre prospective cohort study; future work will be devoted to testing and verifying the application of the DL-based model in a clinical environment and obtaining more imaging data to support further research.

## Supplementary Information

**Additional file 1**. ShuffleNet original model.

**Additional file 2**. ShuffleNet v2-Attenion Pattern Graph.

**Additional file 3**. The structure of ShuffleNet v2-Attenion Data Augmentation.

**Additional file 4**. Database of this deep learning model(github).

## Data Availability

The Chest X-ray imaging datasets used and/or analyzed during the current study are not publicly available due to the policies of the fund funded institutions, the research data may be uniformly disclosed after whole research completed but are available from the corresponding author on reasonable request.The deep learning model online direct link of data is available at https://github.com/HantianDong1988/Pneumoconiosis-Clinical-CXR-imaging-feature-research.
