# Supplementary figures and images for: Use data augmentation for a deep learning classification model with chest X-ray clinical imaging featuring coal workers' pneumoconiosis
Source: BMC Pulm Med. 2022 Jul 15;22:271. doi: 10.1186/s12890-022-02068-x (PMC9284687; doi:10.1186/s12890-022-02068-x)

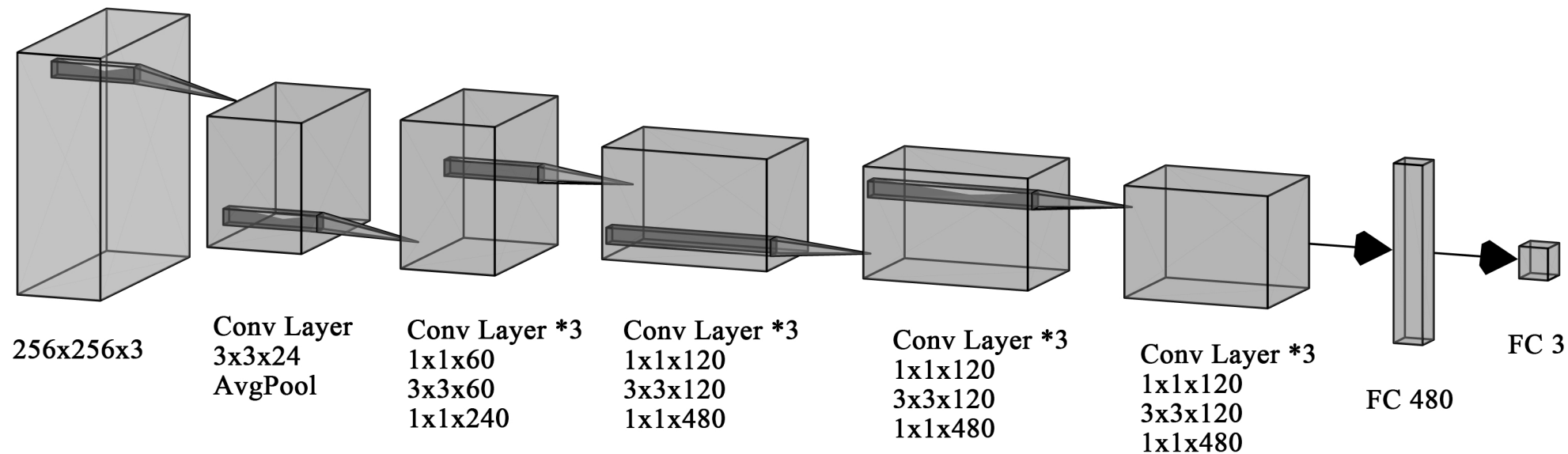

Additional file 1. SuffleNet original model

Supplement: Supplementary file 1 — Additional file 1. ShuffleNet original model. [file 12890_2022_2068_MOESM1_ESM.pdf]

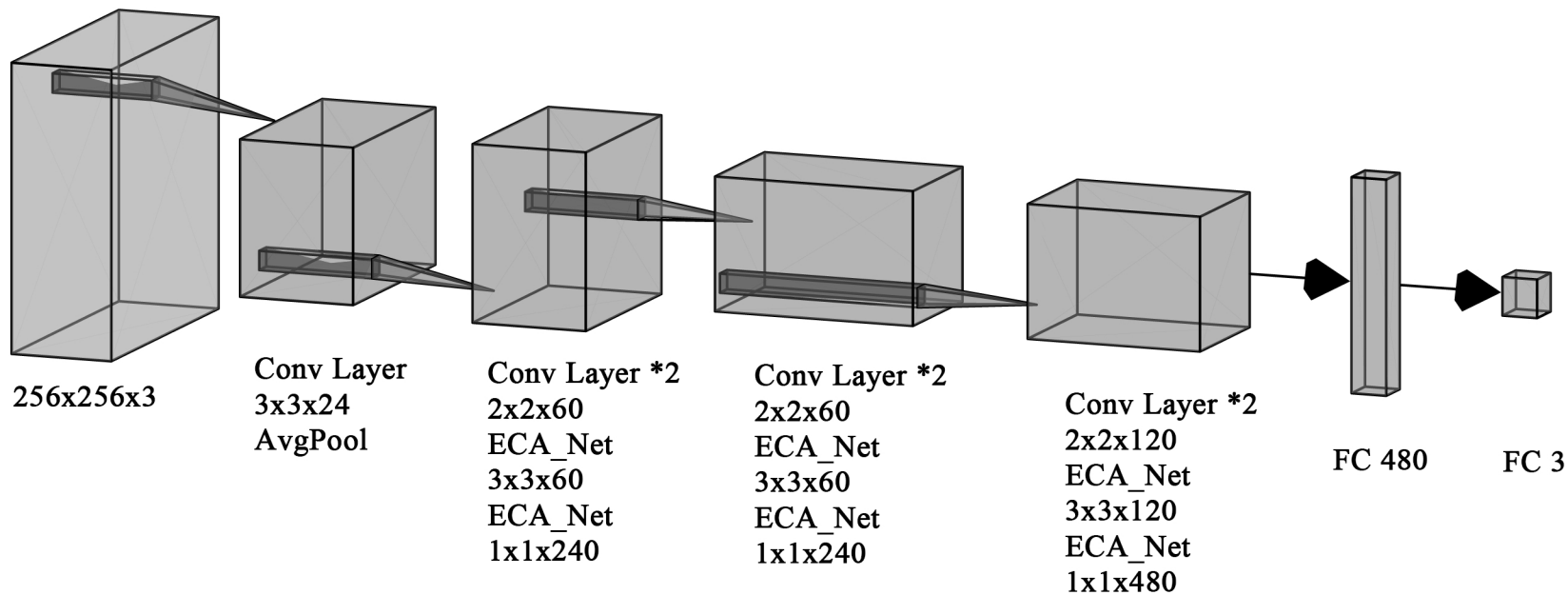

Additional file 2. ShuffleNetv2-Attention Pattern Graph

Supplement: Supplementary file 2 — Additional file 2. ShuffleNet v2-Attenion Pattern Graph. [file 12890_2022_2068_MOESM2_ESM.pdf]

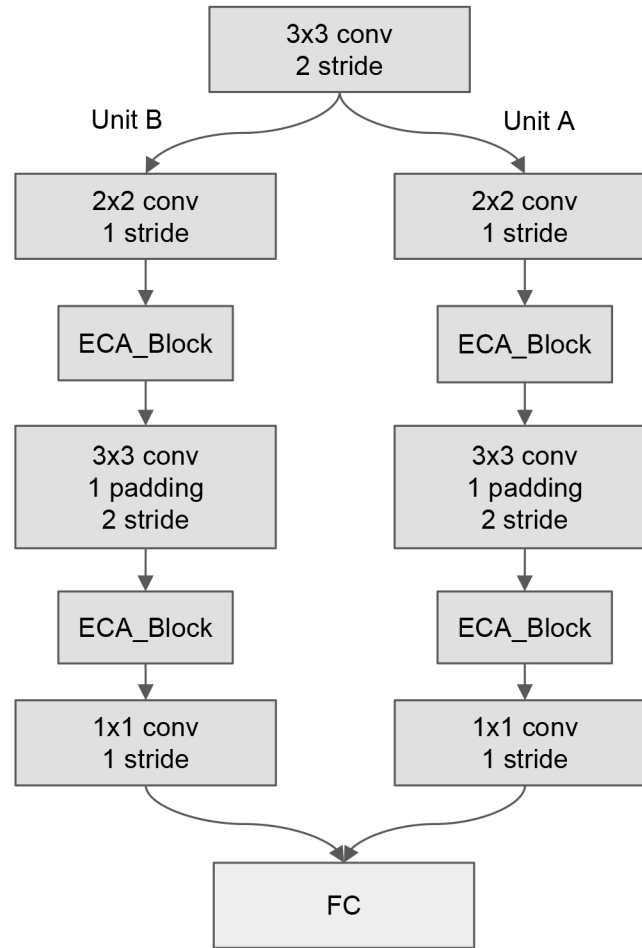

**Additional file 3. The structure of ShuffleNetv2-Attention Data Augmentation**

Supplement: Supplementary file 3 — Additional file 3. The structure of ShuffleNet v2-Attenion Data Augmentation. [file 12890_2022_2068_MOESM3_ESM.pdf]
