# Supplementary material for: Use data augmentation for a deep learning classification model with chest X-ray clinical imaging featuring coal workers' pneumoconiosis
Source: BMC Pulm Med. 2022 Jul 15;22:271. doi: 10.1186/s12890-022-02068-x (PMC9284687; doi:10.1186/s12890-022-02068-x)
Supplement: Supplementary file 4 — Additional file 4. Database of this deep learning model(github). [file 12890_2022_2068_MOESM4_ESM.docx]

https://github.com/HantianDong1988/Pneumoconiosis-Clinical-CXR-imaging-feature-research
